# Multimodal analysis in symptomatic MIDD-associated retinopathy. A case report and literature review

**DOI:** 10.3205/oc000231

**Published:** 2023-12-12

**Authors:** Katarzyna Chwiejczak, Daniel Byles, Paul Gerry, Hirut Von Lany, Anastasia Tasiopoulou, Andrew Hattersley

**Affiliations:** 1 Sheffield Teaching Hospitals NHS Foundation Trust, Sheffield, United Kingdom; 2 The University of Sydney, Australia; 3 West of England Eye Unit, Royal Devon University Healthcare NHS Foundation Trust, Exeter, United Kingdom; 4 Neurophysiology Department, Royal Devon University Healthcare NHS Foundation Trust, Exeter, United Kingdom; 5 Athens Eye Center, Athens, Greece; 6 The MacLeod Diabetes and Endocrine Centre, Royal Devon University Healthcare NHS Foundation Trust, Exeter, United Kingdom; 7 College of Medicine and Health, University of Exeter, United Kingdom

**Keywords:** maternally inherited diabetes and deafness, noninsulin-dependent diabetes mellitus with deafness, pigmentary retinopathy, mitochondrial disease, OCT, OCT-angiography, multimodal imaging, fundus autofluorescence, retinal ganglion cell layer, diabetes mellitus

## Abstract

**Purpose:**

To present results of contemporary multimodal ophthalmic imaging in a case of maternally inherited diabetes and deafness (MIDD) and a literature review of MIDD.

**Methods:**

A case of a 47-year-old female with diabetes mellitus, severe insulin resistance, familial lipodystrohy, deafness and increasing problems with vision is reported. A full ophthalmic examination was done, including best corrected visual acuity (BCVA, LogMAR), funduscopy, and imaging studies: optical coherence tomography (OCT), OCT angiography (OCT-A), fundus autofloresence (FAF), visual fields (HVF) 10-2 , electrophysiology (EP) and genetic testing were performed. Literature available on the topic was reviewed.

**Results:**

BCVA was 0.06 LogMAR in the right eye and 0.1 LogMAR in the left. Funduscopy revealed atrophy (AT) and pigmentary changes but no diabetic retinopathy. HVF confirmed corresponding defects. The imaging and diagnostic tests showed the following abnormalities: FAF: hypoautofluoresence in areas of AT and mottled appearance in the macular and peripapillary area; OCT: attenuation of outer retinal layers and retinal pigment epithelium (RPE) in the AT; OCT-A: thinning of the deep capillary plexus and choriocapillaris; EP: abnormalities on full field electroretinogram (ERG), 30 Hz flicker and single cone flash response; multifocal ERG: reduced responses; genetic testing: A-to-G transition mutation at position 3243 of the mitochondrial genome, typical for MIDD. After one year OCT ganglion cell analysis showed loss of thickness.

**Conclusions:**

Genetic testing should be considered in diabetic patients with pigmentary retinopathy. Imaging studies and diagnostic testing showed structural and functional retinal changes, confined to the macula and progressive in nature.

## Introduction

Maternally inherited diabetes and deafness (MIDD, Online Mendelian Inheritance in Man no. 520,000 [1]; other name: noninsulin-dependent diabetes mellitus with deafness) was first described in 1992. It belongs to mitochondrial diseases, which are caused by mutations in mitochondrial DNA (mtDNA). More specifically, the mutation responsible for MIDD is a single point mutation, A-to-G transition in position 3243 encoding tRNA for leucine (m.3243A>G) [2]. The term ‘mitochondrial disease’ describes a clinically heterogeneous group of conditions in which the tissues and organs that are most often affected are those with the highest energy demands as a result of mitochondrial dysfunction. The most common involvement includes the central nervous system, muscles, retina, kidneys and pancreas. They can be caused either by mutation in nuclear mitochondrial genes and follow Mendelian inheritance patterns, or be associated with mtDNA defects and be inherited maternally. In the latter case phenomenon of heteroplasmy (coexistence of a mix of mutant and wild-type mtDNA molecules) is very characteristic and may determine the phenotype, although the correlation is not always very clear. For these reasons diagnosing patients with mtDNA mutations can often be challenging [3], [4], [5], [6].

Mutation 3243A>G has been found as responsible for a few syndromic conditions: MELAS (mitochondrial myopathy, encephalopathy lactic acidosis and stroke like episodes), MERFF (myoclonic epilepsy, associated with ragged red fibers), chronic progressive external ophthalmoplegia, Kearns-Sayre syndrome, Leigh syndrome and MIDD [7]. Diabetes mellitus is the most common endocrinopathy found in mitochondrial diseases with m.3243A>G being the most prevalent mutation amongst this subgroup of patients [5]. This mutation was reported in 0.13–2.9% of all the diabetic population with noticeable variability between different countries [8], [9], [10], [11].

Diabetes in MIDD usually has insidious onset, but can present acutely in 20%. In most patients islet cell or GAD (glutamic acid decarboxylase) antibodies are absent. The mean age at diagnosis is 37±11 years, ranging from 11 to 68 years. The diabetes arises from damage of B-cells and progresses quickly to insulin dependence, and even though insulin resistance is not typical, it can occur as well [4], [5], [7]. 

A characteristic ophthalmic finding, present in 86–87% of patients with m.3243A>G, is mitochondrial retinopathy. Clinical appearance can range from granular salt-and-pepper macular and peripapillary pattern dystophy to chorioretinal atrophy resembling geographic atrophy (GA) in age-related macular degeneration (AMD). Phenotypes of mitochondrial retinopathy can be classified depending on the type and extent of retinal changes. It is a progressive condition and not stationary. Regarding the symptoms, they are more common in advanced cases and include reduced night vision, scotoma or reduced VA (observed in 61–69% of patients in this heterogenic population) [12], [13]. In MIDD, specifically in the majority of reported cases (about 77%), the patients are asymptomatic [14], [15]. Also, it was observed that the frequency of abnormalities typical for diabetic retinopathy (DR) in those patents was lower compared to type 2 diabetes, but the difference was not statistically significant [14], [16]. 

## Case description

We would like to present a case report of a 47-year-old female with diabetes mellitus diagnosed at the age of 26 and severe insulin resistance, familial lipodystrophy, sensorineural hearing loss and acanthosis nigricans who was referred to the West of England Eye Unit (WEEU), Royal Devon University Healthcare NHS Foundation Trust (formerly Royal Devon and Exeter NHS Foundation Trust), by her optician because of increasing problems with vision, particularly missing some objects in the visual field. Previous genetic testing excluded PPARG (Peroxisome Proliferator Activated Receptor Gamma) and LMNA (Lamin A/C) gene mutations that cause familial partial lipodystrophy. There was no significant family history of diabetes, deafness, lipodystrophy or ophthalmic conditions. Previous ocular history was unremarkable.

Full ophthalmic examination was performed. We tested best corrected visual acuity (BCVA) with ETDRS charts at 3 meters. Colour vision was assessed using Ishihara charts. Adnexa and pupillary reactions were assessed. Slit lamp examination of the anterior segments was performed and intraocular pressure was measured by means of Goldmann applanation tonometry. Both pupils were dilated with guttae tropicamide 1.0% and phenylephrine 2.5%. Slit lamp funduscopy using 90D and WideFileld lenses (Volk) was performed. Imaging studies included optical coherence tomography (OCT, macular cube 512x128) and OCT angiography (OCT-A, 8x8 mm scans) (OCT Cirrus 5000^®^, Carl Zeiss Medic Inc.), fundus photography, blue light fundus autofluorescence (FAF) (λ=488 nm) (Spectralis^®^, Heidelberg Engineering GmbH). Automated perimeter was conducted at separate appointment and with undilated pupils using Humphrey visual fields (HVF) 10-2 (Carl Zeiss Medic Inc.). Electrophysiology (EP), including pattern, full field and multifocal electroretinogram (ERG) and visually evoked potentials, were performed in adherence to the International Society for Clinical Electrophysiology of Vision (ISCEV) standards (Espion: 676-676, Software version: 6.2016.326.56). Consent for use of anonymized data and imaging for research purposes was obtained from the patient.

**Figure 1:**
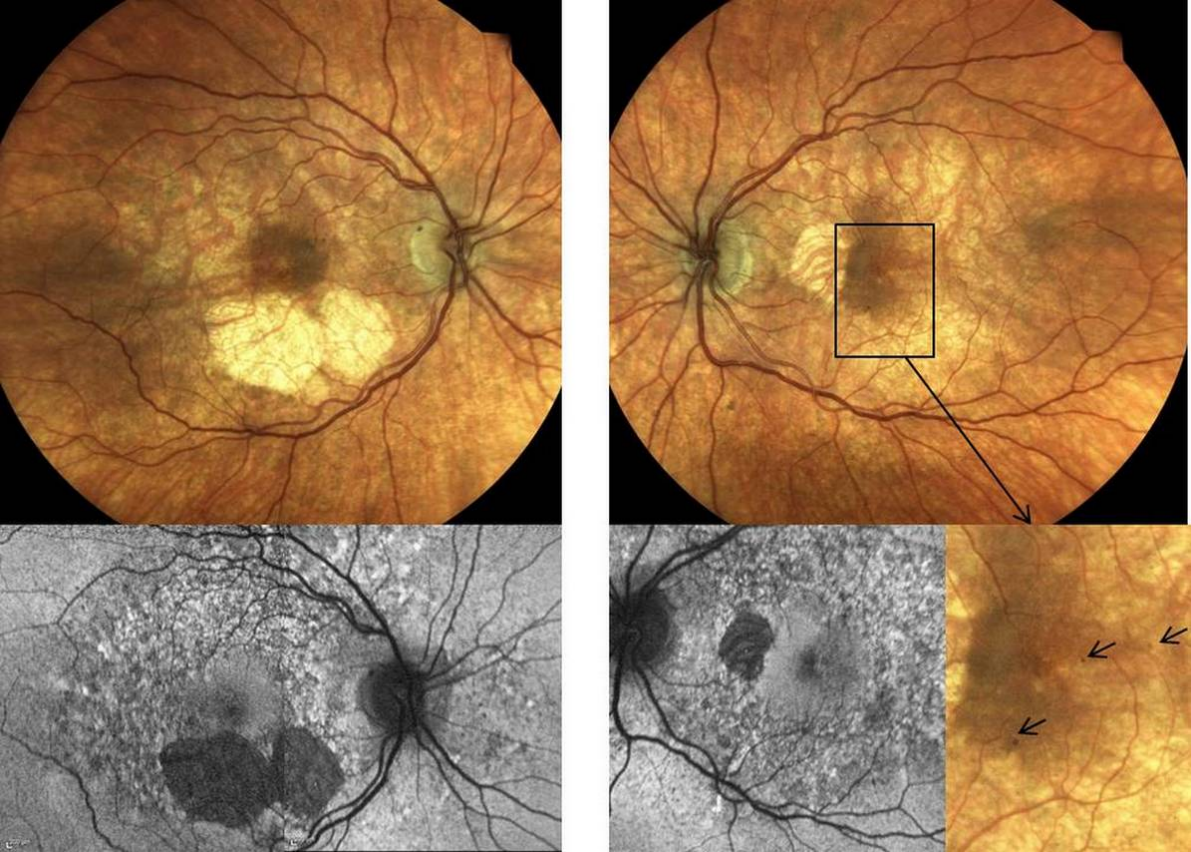
Figure 1 Colour fundus photographs (top row): overall increased translucence of the RPE with visible choroidal vasculature. Area of atrophy in the inferior macula of the right eye is more extensive than in the left eye, where the location is nasal. Also, in the right eye the atrophy is closer to the foveola as compared with the left side. Very few microaneurysms/dot haemorrhages in the macula of both eyes, better visible under magnification (bottom right, arrows). Blue light autofulorescence image (bottom row; right eye: composite of 2 images of the macula and optic disc) clearly shows the extent of the retinal atrophy and reveals the true extent of the retinal pathology with characteristic “salt and pepper” mottled hyper- and hypoautofluoresence beyond vascular arcades and in nasal peripapillary area.

At first presentation in January 2019 her BCVA was 0.06 LogMAR in the right and 0.1 LogMAR in the left eye. Adnexa, ocular motility, pupillary responses, colour vision, intraocular pressure and anterior segments appearance were normal. Dilated funduscopy showed signs of mild diabetic retinopathy: a few microaneurysms and dot haemorrhages in the macular area. The retina appeared mildly thinned and a remarkable finding were bilateral atrophy (AT) and pigmentary changes more pronounced in the right macula. The foveola was spared in both eyes, but in the right eye atrophic changes encroached inferior foveal area (Figure 1 (Fig. 1)). The changes resembled GA seen in AMD. However, taking into consideration the patient’s young age, AMD was excluded as a diagnosis and further investigations were conducted. 

**Figure 2:**
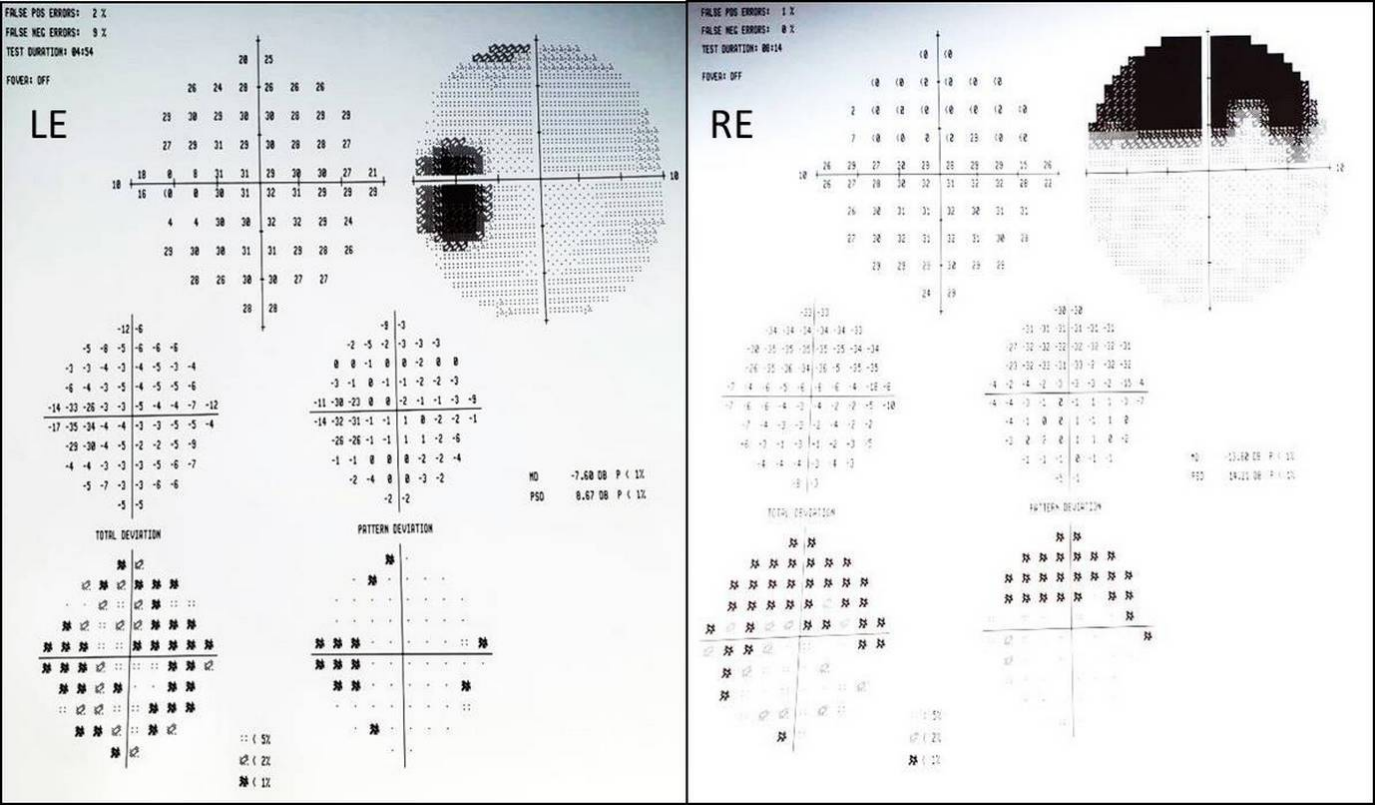
Visual fields: Humphrey 10-2 showing defects consistent with atrophy

Fundus autofluorescence (FAF) (Figure 1 (Fig. 1)) revealed hypo-autofluoresence (AF) corresponding to AT and mottled hypo- and hyper-AF in the macular and peripapillary area. The area involved with retinal changes was more extensive on FAF compared to clinical examination. HVF (Figure 2 (Fig. 2)) confirmed reduced sensitivity corresponding to the area of AT.

**Figure 3:**
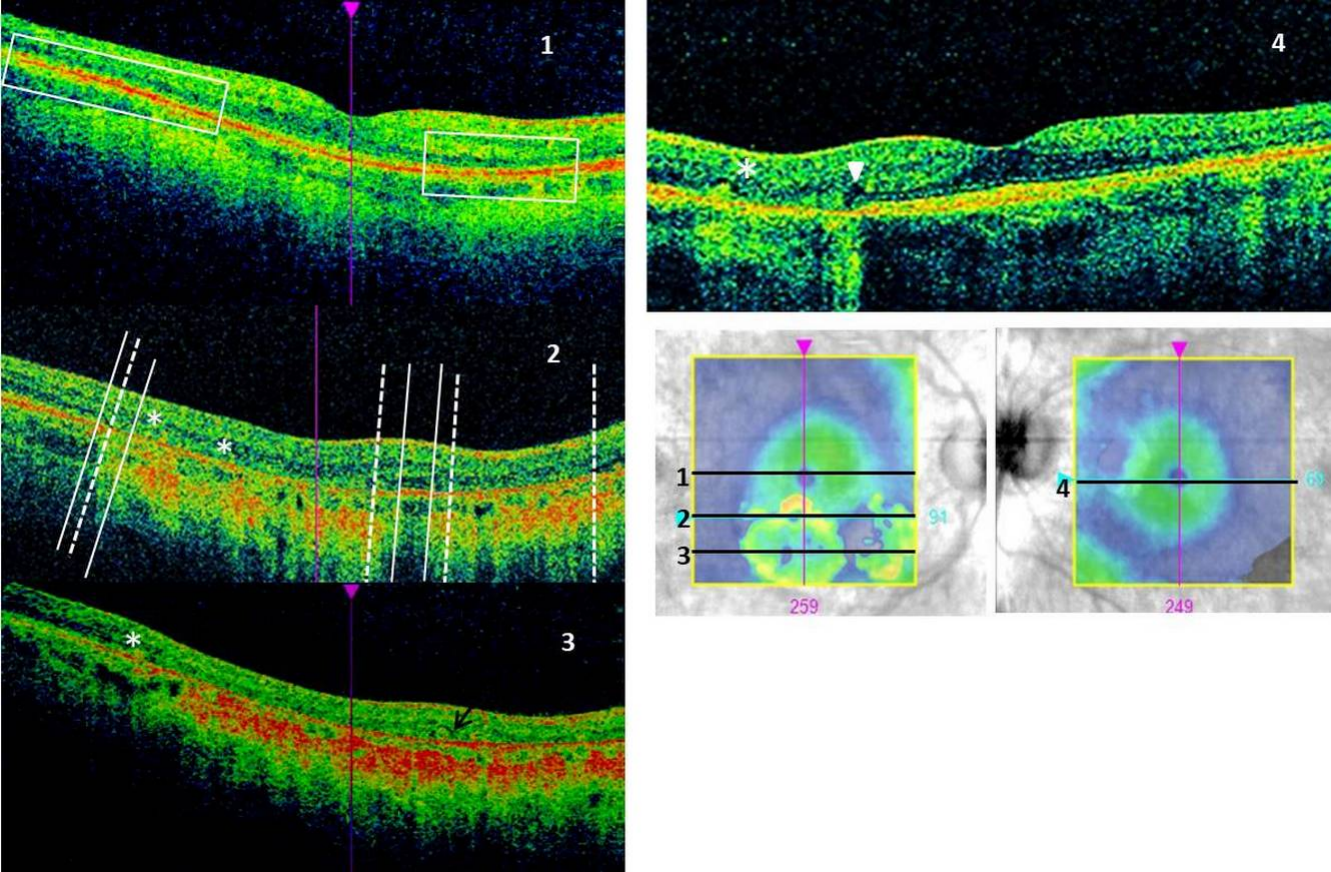
OCT scans Right eye: 1–3 show slices from different levels: 1. foveolar: normal retinal profile, no cystoid macular oedema, but intraretinal hyperreflective foci visible as well as reduced thickness of outer retina and granular irregularity at its level (rectangular frames); 2. superior part of the atrophy: two atrophic areas separated with preserved retina. Extent of RPE atrophy (solid lines) is larger than outer retinal loss (dashed line); 3. central part of the atrophy: loss of RPE and outer retinal layers. Outer retinal tubulation marked with an arrow. Left eye: 4. nasal area of atrophy with visible hyporeflective wedge (arrowhead). On images 3 and 4 subretinal hyperreflective material is marked with asterisks. Also increased transmission to the choroid is present on the images along atrophic regions.

On OCT (Figure 3 (Fig. 3)) we noted loss of retinal pigment epithelium (RPE) and outer retinal layers (ORL) (photoreceptor-ellipsoid, external limiting membrane and outer nuclear layer), smaller in size compared to RPE loss, in the area of atrophy, with concomitant increased signal transmission to the choroid. We also found outer retinal tubulation and hyporeflective wedges. Outside atrophic areas granular irregularity at the level of the outer retina was visible as well as hyperreflective intraretinal foci. Also thinning of outer retina within atrophic areas was noticeable in the perifoveal macula. Average retinal thickness and cube volume of the scans was reduced at 246 and 226 µm and 8.9 and 8.1 mm^3^ in the right and left eye, respectively (normal range: 257.1–295.0 µm and 9.26–10.62 mm^3^) [17]. 

**Figure 4:**
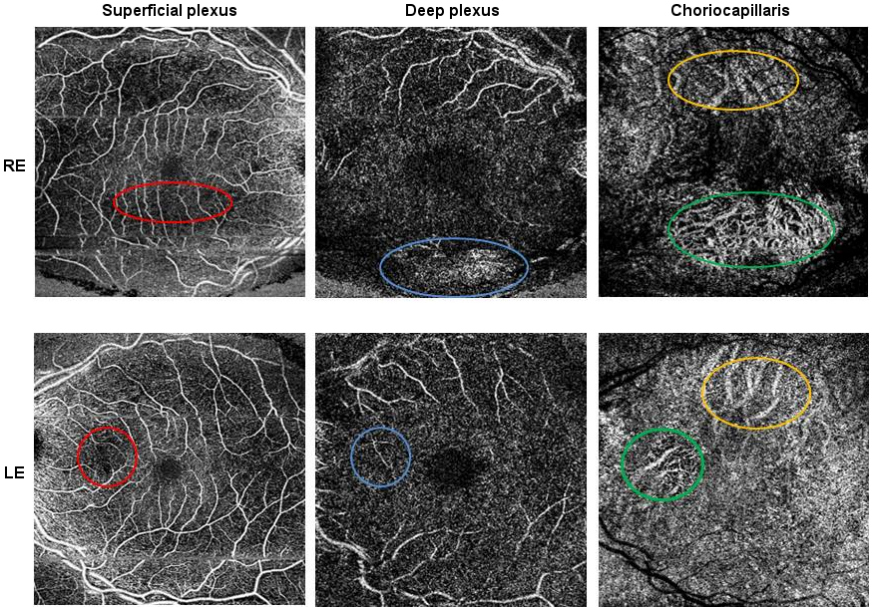
OCT-A Superficial plexus: rarefaction in the perifoveal region visible as a darker area surrounding the fovea, in the right eye also in the inferior fovea and in the left eye in nasal macula (red circle) in the location of atrophy. Deep plexus: transmission (right eye) and projection (left eye) artifacts in the area of atrophy (blue circle). Choriocapillaris: thinning with increased transmission form the deep choroid in the area of atrophy (green circle) as well as outside (orange circle). RE=right eye; LE=left eye

OCT-A (Figure 4 (Fig. 4)) showed minimally enlarged foveal avascular zone and thinning of deep plexus and choriocapillaris in the area of atrophic changes. The superficial plexus was relatively well preserved with some rarefaction, mainly in the area of atrophy and also outside of the atrophic areas in the right eye. In addition, there were a few microaneurysms within the superficial plexus in both eyes.

**Figure 5:**
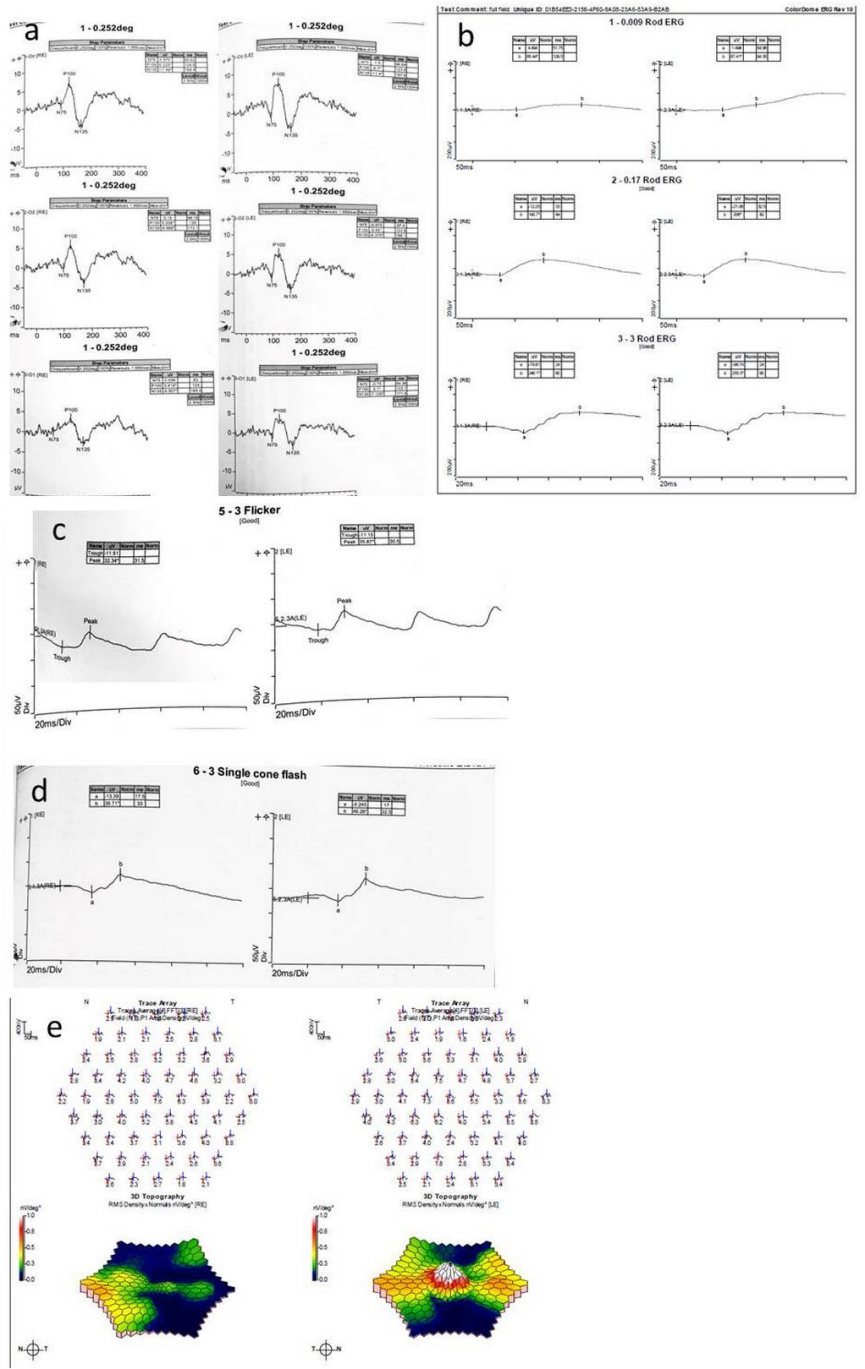
Electrophysiology results a: delay of the P100 wave for small check size; b: full field ERG: low amplitude scotopic B waves; c: 30 Hz flicker: an increased implicit time/latency; d: single cone flash: increase latency and reduced amplitude; e: multifocal ERG: reduced responses, with no foveal peak on the right and only a small one on the left

Electrophysiology findings confirmed abnormalities associated with central macular dysfunction (Figure 5 (Fig. 5)). VEP (visual evoked potentials) testing to small and large check size for each eye showed delay of the P100 wave for small check size only. Pattern ERG was normal. The full field ERG showed low amplitude scotopic B waves. The 30 Hz flicker showed an increased implicit time/latency at 31.5 milliseconds on the right eye and 30.5 on the left. The single cone flash response also showed an increase latency and was below normal in amplitude. Multifocal ERG demonstrated reduced responses, with no foveal peak on the right and only a small one on the left. 

The combination of the retinal findings, diabetes and deafness led to suspicion of a monogenic syndrome, MIDD. Genetic testing showed an A-to-G transition mutation at position 3243 of the mitochondrial genome. Since there was no family history of a similar condition, the above mentioned mutation probably represents a new one. 

**Figure 6:**
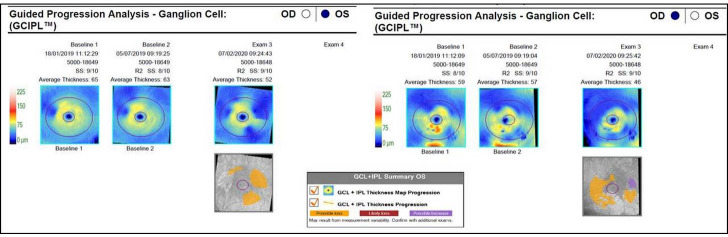
Ganglion cell and internal plexiform layer change during the observation period showing thinning in both eyes

The patient was followed up for a year. BCVA remained excellent: 0.0 LogMAR in the right eye and –0.1 LogMAR in the left. OCT retinal ganglion cell (RGC) analysis showed loss of combined RGC and internal plexifom layer (IPL) thickness in both eyes (Figure 6 (Fig. 6)). In the right eye we observed a reduction from 59 to 49 µm and in the left from 65 to 52 µm. Macular volume and average thickness as well as retinal thickness in all quadrants remained relatively unchanged in the course of observation.

## Discussion

One of the features consistently associated with A3243G mutation is mitochondrial retinopathy. The appearance on funduscopy and severity of symptoms can vary between individuals. Two types of lesions were described: 1. speckled or patchy (“salt and pepper”) hyperpigmentation and 2. atrophy with loss of retinal pigmentation along with retinal atrophy particularly in the posterior pole [14], [18], [19]. Other types of mitochondrial diseases can also be linked to retinal changes. A recent classification by Birtel et al. distinguishes 3 types of retinopathy across various mitochondrial conditions based on extent and pattern of retinal deposits:

1. No visual problems, mild, focal pigmentary abnormalities and corresponding increase or decrease in AF
2. Multifocal faint white-yellowish or hyperpigmented subretinal deposits, hyperautofluorescent dot or fleck-like on AF, pigment changes, chorioretinal atrophy (hypo-AF); symptoms are present in more advanced lesions
3. Granular pigmented fundus alterations extending beyond arcades corresponding to a granular AF pattern, with accompanying atrophy in more advanced cases

In some patients identification of these retinal changes can be crucial for diagnosis of mitochondrial diseases, which otherwise can be challenging. In this study all patients with the type 2 carried m.3243G>A mutation. This specific retinal phenotype is characteristic for the mutation associated also with MIDD and is consistent with the findings in our patient. If present, it can indicate the type of mutation and the diagnosis, as it happened in our experience [13].

De Laat et al. describe 4 grades of severity [12]:

1. Discrete pigmentary abnormalities
2. White-yellowish or hyperpigmented subretinal deposits
3. Chorioretinal (“geographic”) atrophy outside of the fovea
4. Profound chorioretinal atrophy affecting central fovea with decrease in visual acuity

Both studies reported progressive changes in longitudinal data in form of new as well as enlargement of pre-existing atrophy. The proportion of symptomatic cases varies between reports: overall in mitochondrial retinopathy symptomatic cases (reduced dark adaptation, loss of visual field, reduced visual acuity) constitute 48–67% [12]. In MIDD, symptoms were often minimal or absent (57–80% of patients), but in some patients visual problems can occur as a result of photoreceptor loss [7], [14], [15], [20]. The patient we present belongs to the rare group of symptomatic mitochondrial retinopathy with functional impairment confirmed by HVF testing and electrophysiology. 

In our patient FAF was particularly valuable for assessment of the real extent of RPE changes. The pattern visible on FAF was used for the above mentioned classification systems. Findings by Rath et al. coincide with abnormalities described by the other groups, while focusing on features of FAF in MIDD retinopathy. The most common phenotype was perifoveal atrophy that would extend during follow up with foveal sparing. Decreased AF was present in areas of atrophy, while the pale deposits revealed increased AF. Similar to our patient speckled AF appearance was not evident on clinical examination. FAF not only reveals the true extent of retinal changes, but also shows the characteristic pattern of retinopathy for mitochondrial disease [19]. This way it can support the diagnosis of MIDD even in asymptomatic individuals [12], [13], [21], [22].

A group from London, Müller et al., analyzed results of FAF and assessed its prognostic value. They confirmed that typically the mitochondrial retinopathy spares central 10–20° of the retina and affects the area with highest rod density. It seems that the rods could be more susceptible to damage, hence some patients experience difficulties with dark adaptation and night vision. In our patient the atrophy encroached the inferior fovea, the reason why she developed partial loss of visual field. This type of presentation might be associated with early visual impairment, earlier age of onset and higher progression rate. This group further concluded that FAF imaging allows for monitoring of progression (mean increase 2.33 mm^2^/year) and could serve as a tool for outcomes should any treatment become available. The group showed also that the main prognostic factors for progression were sex, age, and number of atrophic foci [20], [23]. A study from Australia supports the importance of FAF in diagnosis and monitoring of MIDD retinopathy [24]. In our patient FAF was only performed once, so we were unable to comment on the progression rate. 

Electrophysiology results are not consistent and vary significantly between individuals [25]. In the series by Birtel et al. on full field ERG, scotopic and photopic responses were reduced in type 3 mitochondrial retinopathy, which extends beyond the macula [13]. Abnormal electrooculogram (EOG) can indicate abnormalities in the RPE/photoreceptor complex [15]. This study was not performed in our patient. Reduced dim flash scotopic b-wave ERG was another frequent finding, suggesting dysfunction of predominantly rod photoreceptors and RPE [26]. Full field ERG is generally normal, but multifocal ERG (mfERG) can better detect macular abnormalities and show reduced responses. It was suggested that reduced peak amplitudes with normal implicit times in the mfERG indicate localized loss of function and damage to the cone photoreceptor outer segments or cone photoreceptor loss. Findings in our patient suggest involvement from both types of photoreceptor cells and mainly macular involvement. Pattern ERG was normal, indicating relatively preserved central macular function [25].

OCT findings in our patient were in line with previously described reports. Mitochondrial diseases affect tissues with high metabolic demand, hence RPE and photoreceptor layer undergo damage early in the course of the disease. Histopathological findings and data from clinical studies (FAF and OCT analysis) suggest early RPE involvement, with changes in apical structure of RPE cells and loss of melanin. However, in some cases – as described by Birtel et al. [13] – the photoreceptors seem to undergo damage at an earlier stage. These structures are very closely related – both anatomically and metabolically – and exact chronology is difficult to determine [12], [13]. Müller et al. proposed that the course of progression starts at ellipsoid zone first with subretinal deposits, later external limiting membrane loss and subsequent RPE integrity disruption with outer nuclear layer thinning and photoreceptor outer segments shortening completing formation of the atrophy. They hypothesize that subretinal deposits could represent early RPE dysfunction and/or increased outer photoreceptor segments shredding. Ellipsoid is affected first as the most energetically demanding and packed with mitochondria layer. Focality of these changes might result from heteroplasmy in different areas of the retina [27]. In case series by Tripathy et al. the findings are very similar to our patient [28]. We did not observe pseudocysts, but we were able to identify outer retinal tubulation – similar as described in AMD over areas of fibrosis or RPE dysfunction and in other conditions with associated outer retinal damage [28], [29], [30]. Hyporeflective wedges are found at the border of retinal atrophy and are thought to derive from axonal swelling or interaxonal edema within the outer plexiform layer (OPL). Their location right above RPE atrophy seems to confirm that RPE thinning precedes ORL loss [27].

Our OCT-A images were to some degree obscured by motion artifacts, which made the interpretation more challenging. Nevertheless, this imaging showed structural abnormality of the retinal blood circulation in the course of atrophy. Tripany et al. analyzed the results of OCT-A imaging and similarly as in our case they found choriocapillaris rarefaction, even in areas with no evident atrophy [28]. Loss of choriocapillaris outside the area of atrophy could suggest that this layer can be affected early in the course of the disease. A group from Tunisia reported foveal avascular zone enlargement and areas of retinal capillary rarefaction in superficial and deep capillary plexuses [31]. In our case deep plexus was difficult to analyze because of transmission artifact related to thinned retina. Whether this is just purely segmentation defect or an indicator of true loss of vascular structure at this level would need to be determined by further studies. Some laboratory studies show that vascular changes have a prominent role in the process of retinal atrophy. A group from University of Murcia showed that disruption of retinal circulation might be associated with remodeling of the outer retinal plexus, so that it forms “subretinal vascular complexes” with abnormal structure [32]. Even though we only present one case and the testing was made at only 3 time points, it might be that this observation is not a coincidence. The group from Murcia showed in an animal model that ganglion cell layer undergoes death as a result of photoreceptor degeneration in acquired and congenital conditions, like AMD and retinitis pigmentosa. They observed impaired axonal transport in RGCs and death that happened as a late stage in the course of retinal photoreceptor dystrophy [32]. Also in clinical studies loss of RGC layer was observed on OCT scans of patients with geographic atrophy in advanced stages [33], [34]. We suggest that a similar secondary process might also happen in the course of MIDD-associated mitochondrial retinopathy. Further data is required to verify this finding. 

There are suggestions that presence of m.3243A>G might reduce prevalence of DR. In a large prospective case-controlled multicenter study by the Mitochondrial Diabetes French Study Group prevalence of DR (as defined by the Early Treatment Diabetic Retinopathy Study (ETDRS) severity scale, i.e. presence of microaneurysms, retinal hemorrhages, cotton wool spots, intraretinal microvascular abnormalities, hard exudates, venous beading, or new vessels) was significantly lower in MIDD population compared to diabetic patients from the control group even after adjustment for HbA1c and systolic blood pressure or the presence of or no hypertension. Despite a mean duration of diabetes of 12 years the patients exhibited a low frequency (8%) of DR. Protection against DR might be related to retinal dysfunction and decreased retinal metabolism and oxygen demand. Also, mitochondrial dysfunction might result in reduction in superoxide production, thus preventing vascular wall damage [35]. Other reports presented similar perspective, supporting reduced frequency of DR in patients with MIDD [7], [20], [36], [37]. There are some publications which disagree with this statement, but they describe lower numbers compared to the French group [15], [38]. Our patient had only a few microaneurysms and dot haemorrhages despite poorly controlled diabetes (HbA1C around 80 mmol/mol) and a history of over 20 years of diabetes. 

In our case, ophthalmic examination turned out to be crucial for the diagnosis. We agree with other authors that patients with diabetes and/or deafness and retinal pigmentary changes should undergo genetic testing for mitochondrial diseases [15], [18]. Patients with A3243G mutation are often asymptomatic or with mild symptoms so routine testing and diabetic screening might be the first examination where the retinal changes are noted. It would be desirable to make screeners aware of the problem, so that patients can be referred for further investigations despite not having any changes related to DR. In the era of increasing presence of artificial intelligence use in diabetic screening this is yet one more variable that should be included in the machine learning [27].

Current progress in gene therapy for treatment of retinal dystrophies, including registered treatment for Leber congenital amaurosis (Luxturna^®^), gives hope to patients affected and imposes further studies on those conditions [39]. Mitochondral dystrophies usually affect multiple organs and systems. In those cases retinal dystrophy can potentially serve for identification of patients and as parameters for clinical endpoints [13].

In summary, we presented an interesting case of a symptomatic patient with retinopathy related to MIDD. We used comprehensive testing and imaging studies to show anatomical and functional impairment. We were able to show retinal changes characteristic of the condition using a wide spectrum of techniques, and to our knowledge we were the first to perform this broad analysis.

## Conclusions

Mitochondrial retinopathy is a common finding in patients with A3243G mutation. Often patients remain asymptomatic. Thus, in diabetic patients with this finding, even in the absence of symptoms, genetic testing and counseling should be considered. In those patients imaging studies and diagnostic testing show structural and functional changes in the retina, confined to the macula with preserved peripheral retinal function. FAF can reveal retinal abnormalities not visible on clinical examination, which suggests that clinically healthy parts of the retina have in fact impaired RPE metabolism and FAF should be included in ophthalmic testing and follow up in patients with MIDD. We observed abnormalities in the retinal vascular structure and choriocapillaris on OCT-A with the latter being affected early in the course of the condition. Electrophysiology confirmed abnormalities mainly in the macula, with signs of involvement from both cones and rods. For the first time we described loss of RGCs in the course of the disease. Loss of RGC over time might represent a secondary process to photoreceptor loss. 

With the advent of genetic treatments there is continuous need for learning more about the potentially treatable retinal conditions and data from imaging studies are essential for planning and monitoring therapeutic studies.

## Notes

### Competing interests

The authors declare that they have no competing interests.
